# Coronary Bifurcation PCI—Part I: Fundamentals

**DOI:** 10.3390/jcdd12100410

**Published:** 2025-10-16

**Authors:** Sara Pollanen, Rongras Damrongwatanasuk, Ju Young Bae, Jason Wen, Michael G. Nanna, Abdulla Al-Damluji, Mamas A. Mamas, Elias B. Hanna, Jiun-Ruey Hu

**Affiliations:** 1 Temerty Medicine, University of Toronto, Toronto, ON M5S 1A1, Canada; 2 Division of Cardiovascular Medicine, Department of Internal Medicine, University of Louisville, Louisville, KY 40208, USA; 3 Division of Cardiovascular Medicine, Weill Cornell Medicine, New York Presbyterian Hospital, New York, NY 10032, USA; 4 Department of Cardiology, Smidt Heart Institute, Cedars-Sinai Medical Center, Los Angeles, CA 90048, USA; 5 Department of Medicine, Section of Cardiovascular Medicine, Yale New Haven Hospital, New Haven, CT 06510, USA; 6 Cardiovascular Center on Aging, Department of Cardiovascular Medicine, Cleveland Clinic Foundation, Cleveland, OH 44195, USA; 7 Keele Cardiovascular Research Group, Keele University, Newcastle ST5 5BG, UK; 8 Cardiology Department, Lebanese American University, Beirut 1102, Lebanon

**Keywords:** primary coronary intervention, bifurcation lesions, bifurcation stenting, coronary artery disease

## Abstract

Percutaneous coronary intervention (PCI) of bifurcation lesions remain one of the most technically challenging areas in interventional cardiology. Careful planning and execution are needed to preserve main vessel and side branch patency, with evolving evidence guiding the choice between provisional and two-stent strategies, and between individual techniques. This narrative review, which represents the first installment of a two-part series, synthesizes current knowledge on bifurcation PCI, detailing the anatomical classifications, lesion assessment tools, procedural planning, and execution of techniques including T and Protrusion (TAP), double-kissing (DK) crush, mini-crush, culotte, V-stent, and emerging modifications. We contextualize the choice of strategy within lesion complexity, procedural goals, and patient-specific considerations. This review is intended as a visual, practical, technique-focused reference for interventionalists and interventional trainees involved in the management of bifurcation lesions.

## 1. Introduction

Coronary bifurcation lesions account for approximately 20% of all percutaneous coronary intervention (PCI) and represent a technically challenging subset of coronary artery disease [1]. Coronary bifurcation lesions occur adjacent to, and/or involve, the origin of a significant side branch (SB), often supplying a large myocardial region that should be preserved [2,3]. The treatment of these lesions is associated with lower procedural success and higher rate of long-term complications including restenosis and stent thrombosis compared to non-bifurcation PCI [4]. This is due to the need to maintain patency in the main vessel (MV) and SB, and risks of SB occlusion during intervention. Several classifications have been proposed, with the Medina classification being the most widely adopted due to its simplicity. Bifurcation lesions involving the left main (LM) artery are particularly important to distinguish as they are associated with a larger amount of jeopardized myocardial territory, with more than 80% of these cases involving distal bifurcation disease [5,6]. In contrast, non-LM bifurcations, while still clinically important, typically involve smaller myocardial territories and may present different technical challenges [7].

Over time, various bifurcation stenting techniques have been developed and refined, including T-stenting, T-and-protrusion (TAP) stenting, regular crush, mini-crush, double-kissing (DK) crush, and Culotte, T-stenting. A recent network meta-analysis of 21 randomized controlled trials involving 5711 patients found that DK crush had lower MACE compared to regular crush, T/TAP, and Culotte, but there was no difference in cardiac death, myocardial infarction, or stent thrombosis [8]. These findings also underscore ongoing debate about the relative value and safety of each technique. In the first part of this two-part review, “Fundamentals of Coronary Bifurcation PCI”, we review major bifurcation lesion classification systems and procedural strategies, with a special focus on visualization of step-by-step techniques for the practicing clinician. In the companion paper that follows, “Advanced Considerations in Coronary Bifurcation PCI”, we review outcomes associated with different intervention techniques, use of drug coated balloons and pressure wire testing in bifurcation PCI, SB considerations, and an algorithmic approach to selecting the optimal technique for a given patient [9].

## 2. Classification of Bifurcation Lesions

### 2.1. Medina Classification

**Figure 1:**
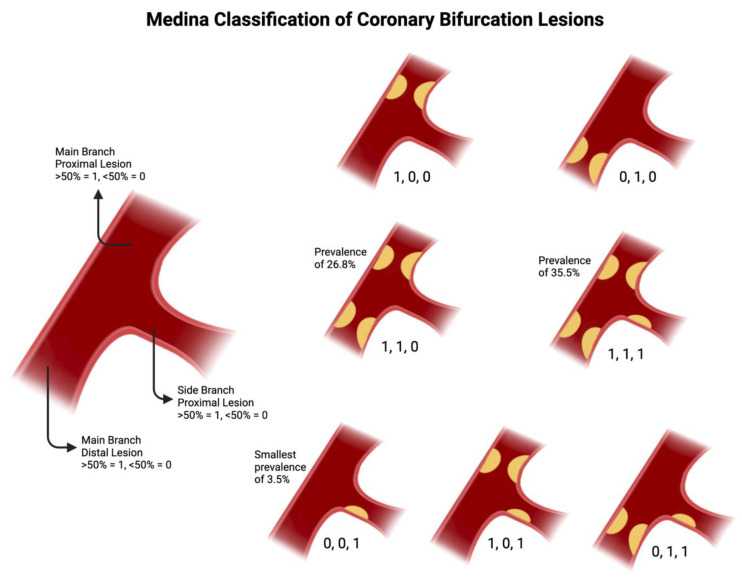
Medina Classification, with prevalence data taken from [14]. Original illustration.

Introduced in 2006 by Medina et al., the Medina classification provides a systematic approach of categorizing coronary bifurcation lesions based on angiographic findings [10]. Due to its simplicity, the Medina classification is the most widely used classification system, and it is endorsed by major organizations such as the European Bifurcation Club [10,11,12]. This classification employs a tripartite numerical system, assigning a binary value (0, 1) to denote presence (1) or absence (0) of significant stenosis (≥50% diameter reduction) in three segments of bifurcation [10]. These segments are the proximal MV (segment proximal to bifurcation), Distal MV (segment distal to the bifurcation) and the SB (bifurcating from the MVl), respectively [10]. Seven total classes can be assigned, with values such as 1,1,1 indicating significant stenosis in all three segments, or 1,0,0 indicating stenosis only in proximal MV [10]. These classes have been used to aid in risk stratification and guide choices between provisional and two-stent strategies [13,14]. Lesions can further be separated into true and non-true bifurcation lesions. True bifurcation lesions often call for more complicated interventions, and are defined as stenosis in both the SB as well as the proximal MV and/or distal MV (Medina 1,1,1; 1,0,1; or 0,1,1) [14]. Among these subtypes, Medina 1,1,1 and 0,0,1 have been associated with increased risk of target lesion failure within one year [12]. The prevalence of coronary bifurcation lesion subtypes varies, with Medina 1,1,1 (35.5%) and 1,1,0 (26.8%) being the most common and Medina 0,0,1 (3.5%) the least common (Figure 1) [14].

### 2.2. ABCD Classification

Recently, the ABCD classification system was proposed to describe LM bifurcation and trifurcation lesions [15] to address several limitations of the Medina classification system, which is not specific for left main (LM) disease [15,16]. Revascularization of the LM coronary artery can be complex, with involvement of the bifurcation in up to 80% of cases [17]. The Medina system does not involve sizing of vessels, which is critical in LM stem disease as the LM, left anterior descending (LAD), and left circumflex (LCx) have a wide range of diameters depending on age, sex, ethnicity, and circulatory dominance [11]. The LAD is often assumed to be the MV; however, in patients with left-dominant coronary anatomy with a LCx that gives rise to the PDA, the caliber of the LCx may be larger, such that it effectively functions as a MV while the LAD functions as a SB. This distinction is not shown by the Medina classification, yet it is relevant when choosing to use a provisional stenting strategy [18]. Finally, Medina was not designed to handle trifurcation lesions, despite the EXCEL trial showing 10% of lesions were distal trifurcation lesions.

**Figure 2:**
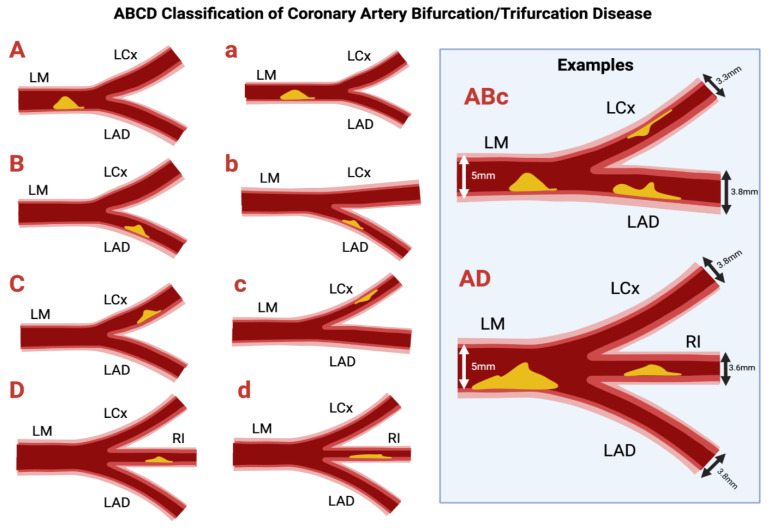
ABCD Classification of bifurcation and trifurcation disease. Original illustration.

In the ABCD system, each artery is assigned a letter if it is significantly stenosed (defined as >70% lumen reduction) (Figure 2). The nomenclature is as follows: A/a → LM artery; B/b → LAD artery; C/c → LCx artery; D/d → ramus intermedius artery (intermediate branch, for trifurcation lesions). If the stenosis is not considered significant, no letter will be assigned. If the vessel diameter is ≥3.5 mm an uppercase letter is used, if <3.5 mm a lowercase letter is assigned. This system allows for 256 ways to describe vessels (i.e., AbC, Bc, bD, etc.), which allows for more opportunity for description than the 7 vessel phenotypes of the Medina classification. However, a key limitation of the ABCD classification lies in its added complexity and the lack of routine adoption in clinical practice. In contrast to the Medina classification, the ABCD system has not been consistently integrated into procedural planning as extending a bifurcation intervention framework to trifurcation lesions can be challenging, as it lacks clearly defined steps and standardization in treatment strategy. Other alternative classification systems to target Medina’s limitations have been developed such as the Movahed classification and qualitative descriptive coding approaches, which will not be covered in the present review.

### 2.3. Finet’s Formula and Bifurcation Vessel Size

**Figure 3:**
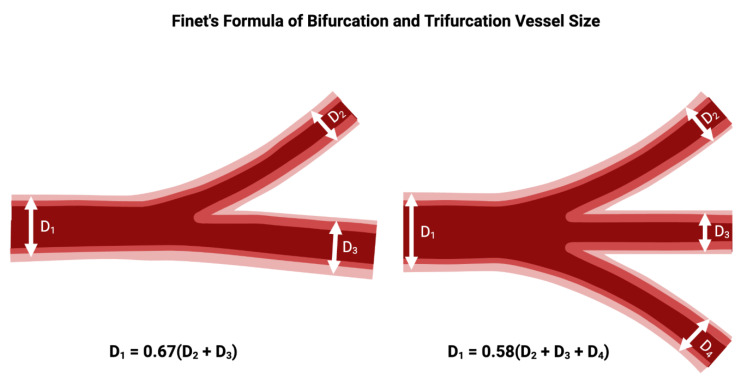
Relationship between diameter of the proximal main branch vessel, distal main branch vessel, and SB vessels in a bifurcation or trifurcation, according to Finet’s formula. Original illustration.

Finet’s formula describes the relationship between vessel diameters in coronary artery bifurcations (Figure 3). The conservation of flow principle states that the total blood flow at the bifurcation point is conserved, meaning that the combined flow from the MV must equal the flow going into both the main and SB. It states that the proximal MV diameter (D1) is approximately 0.678 times the sum of the distal MV diameter (D2) and the SB diameter (D3). For an arterial trifurcation, the diameter of the proximal MV = 0.58 of the diameter of the distal MV and the diameter of the two SB summed together [19]. Notably, this principle of flow conservation was recently demonstrated in vivo in a series of patients undergoing clinically indicated coronary physiologic testing of the left main system. During hyperemia, the sum of flow in the LAD and LCx closely matched flow in the left main coronary artery, confirming the application of Finet’s law of bifurcation geometry [20].

The formula suggests that for the SB to maintain an adequate flow, the diameters of the SB and the MV need to be balanced so that the total flow through both vessels remains optimal. The formula is derived from the Poiseuille’s law of laminar flow, which states that the flow through a vessel is proportional to the fourth power of the radius (or diameter) of the vessel. Therefore, small changes in vessel diameter can lead to large changes in flow. If the SB has a small diameter compared to the MV, the flow through the SB will be lower than the flow in the MV, unless other factors (such as pressure or collateral circulation) compensate for it. This can make the SB more prone to being compromised during coronary interventions. Conversely, when the SB has a diameter similar to or greater than the MV, it is more likely to maintain adequate flow after stenting or balloon dilation [21].

## 3. Technique Fundamentals

### 3.1. Introduction to Provisional Approach

**Figure 4:**
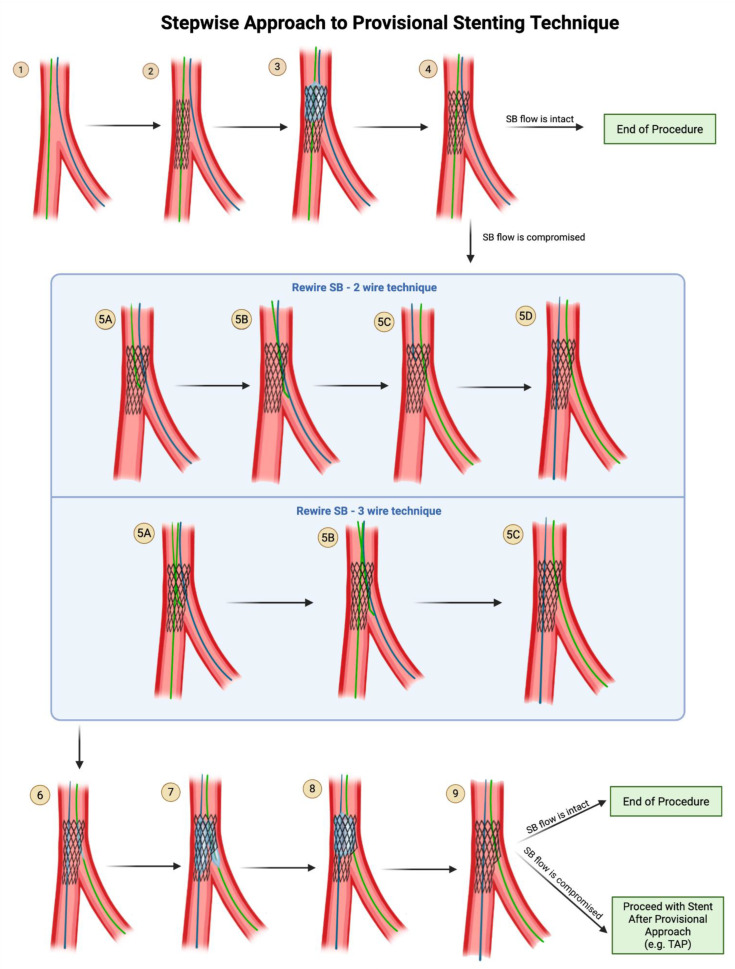
Provisional Stenting Technique. If SB flow is compromised after provisional stenting, the SB can be rewired either in a 2-wire technique or 3-wire technique, followed by ballooning into the SB. Original illustration.

The provisional stenting approach refers to a collection of techniques used in coronary bifurcation interventions where only the MV is stented initially, and the SB is treated only if compromised (Figure 4). Provisional stenting is often preferred in situations where (1) The SB lesion is not severely diseased or is not expected to cause significant ischemia. (2) The SB ostium is not critically involved in the bifurcation lesion, and the MV lesion is the primary concern. (3) The SB has a favorable geometry, meaning the angle between the MV and the SB is not too sharp, reducing the likelihood of occluding the SB when treating the MV. The SB is left unstented unless there is evidence, after initial MV stent delivery, that the SB is significantly compromised and requires stenting [22,23]. The lack of necessity in rewiring and kissing a non-compromised SB is supported by the results of the CROSS, NORDIC 3, and KISS trials [24,25,26]. The EBC recommends proceeding to rewiring the SB to perform KBI only if post-stenting of the MV, SB flow is compromised with TIMI flow <3 or SB stenosis is ≥70%. Note that the cutoff of SB stenosis used in the provisional arms of the NORDIC IV and DK-CRUSH V trials was 75% instead of 70% [27,28].

**Steps of Provisional Technique**
  **Step 1:** Wire the MV and the SB.
  **Step 2:** Advance the stent into the main branch and deploy the stent.
  **Step 3:** Advance a balloon in the proximal segment of MV and perform POT.
  **Step 4:** Remove the balloon from the MV and perform an angiogram to evaluate flow in the SB:
    - If SB flow is intact (TIMI 3), it is acceptable to end the procedure here and remove both wires.
    - If SB flow is compromised (TIMI flow <3 or SB stenosis ≥70%), proceed with rewiring the SB through the distal struts of the MV stent.
**Two Wire Technique for Rewiring SB:**
  **Step 5A:** Withdraw the MV wire back into the MV stent at the level of the SB, with the curved tip of the wire pointing in the direction of the SB.
  **Step 5B**: Enter the SB through the stent struts.
  **Step 5C:** Withdraw the jailed SB wire.
  **Step 5D:** Once the jailed SB wire is withdrawn, advance it to the main branch, using a rotational motion to keep the wire tip free and increase the chances of coursing within the stent lumen.
**Three Wire Technique for Rewiring SB:**
  **Step 5A:** While the main branch wire remains in place, bring a 3rd wire into the MV stent at the level of the SB, with the curved tip of the wire pointing in the direction of the SB.
  **Step 5B:** Enter the SB through the stent struts.
  **Step 5C**: Remove the jailed SB wire out of the body. Note that the MV wire remains in place in the MV.
  **Step 6**: Advance the balloon in the MV and the balloon in the SB vessel.
  **Step 7:** Perform kissing balloon inflation with the SB stent balloon and the main branch balloon. Remove both balloons.
  **Step 8:** (Optional) POT can be applied again after KBI, to prevent oval distortion of the proximal MB by the kissing balloon inflation.
  **Step 9:** Perform an angiogram to evaluate flow in the SB.
    - If SB flow is intact (TIMI 3), it is acceptable to end the procedure here and remove both wires.
    - If SB flow is compromised (TIMI flow <3 or SB stenosis ≥90%), then stenting of the SB may be necessary. Proceed with stent-after-provisional approach, e.g., TAP.

### 3.2. Kissing Balloon Inflation

Bifurcation lesions pose a heightened risk of SB occlusion or stenosis due to mechanisms such as carina or plaque shift, refractory spasm, or ostial dissection. These complications can result in adverse short- and long-term outcomes, including elevated cardiac troponin levels, anginal symptoms, and mechanical dysfunction. In situations where rewiring of the SB is pursued due to SB compromise, the addition of kissing balloon inflation (KBI) has been shown to result in improved minimal stent area at the distal MV, improved SB scaffolding, and lower malapposition in the bifurcation core and distal MV [29]. KBI should be considered as a key step when undergoing two stent strategies for bifurcation lesions and selectively for provisional stent strategy to ensure ostial SB optimization, stent distortion correction, and apposition and patency of main branch stent struts on the ostium of the SB.

**Steps for Kissing Balloon Inflation**

**Step 1**: Place one guidewire in the distal branch and the other guidewire in the SB. In the case of provisional stenting, attempt to rewire the SB through the distal MB stent struts.

**Step 2:** Select two non-compliant balloons based on the distal main branch and SB (see below for sizing guidelines). Frequently, after rewiring through stent struts (e.g., rewiring SB through MB stent struts), a small 2.0 mm (or even as small as 1.0 mm or 1.25 mm in difficult cases) compliant balloon inflation is needed to cross the struts into the SB to allow for subsequent advancement of the properly sized, bulkier non-compliant balloon after serial balloon dilation.

**Step 3:** Advance the distal branch balloon to the stented segment and ensure that the distal balloon marker lands at the tip of the carina and the SB balloon is positioned at the ostium of the SB with the proximal marker at the stent strut borders ensuring minimal overlap between the two balloons at the bifurcation.

**Step 4:** Inflate both balloons in the distal branch and SB simultaneously at 10–12 atmospheres and simultaneously deflate the balloon.

**Step 5:** Remove both balloons.

**Step 6:** Confirm the coronary flow angiographically or using intravascular ultrasound for adequate expansion and opposition.

**How do you choose the size and length of the balloons for kissing balloon inflation?**

Non-compliant balloons should be used to avoid overexpansion at the SB ostium, dissection, or vessel injury while providing adequate main branch expansion. Short non-compliant balloons are commonly recommended for kissing balloon inflation and post dilation in bifurcation PCI because they maintain diameter with rising pressure and reduce “dog boning” which can otherwise promote edge dissection or injury [30]. While randomized trials are not available, observational studies show that NC balloons are associated with a reduced rate of side branch dissection and improved long term outcomes [31,32]. Nevertheless, it should be acknowledged dissections can also depend on sizing and pressure and some operators sometimes may prefer semi- compliant balloons at a delicate side branch ostium. The MV balloon should be selected 1:1 to the diameter of the distal main branch and the SB balloon should be sized 1:1 to the SB reference diameter. The length of the balloon should be kept shorter than the stent to avoid balloon inflation outside of the stent in the main branch and the disease-free areas in the SB.

**POT-side-POT**

Sequential POT-side-POT has been described as an alternative to kissing balloon technique. POT-side-POT is a three-step process: an initial POT on the main vessel, dilation of the side branch, and a final POT to optimize the main vessel stent’s proximal segment [33,34]. However, one trial did not show an advantage of this technique over conventional kissing balloons [35] and there is insufficient data accumulated at this time; the EBC has yet to recommend this as a routine technique.

### 3.3. Proximal Optimization Technique

Proximal optimization technique (POT) is a stent optimization strategy routinely employed after provisional stenting in bifurcation coronary lesions. By optimizing the proximal segment of the MV stent, POT facilitates strut protrusion into the SB, creating a larger strut opening while minimizing carinal shift. This enhances guidewire recrossing and procedural efficiency. Incorporation of POT has been shown to be associated with reduced target lesion failure rate [36].

**Step 1:** Advance a balloon (see below for sizing guidelines) into the MV stent. The distal marker of the balloon should be placed just proximal to the carina.

**Step 2:** Inflate the balloon.

**How do you choose the size and length of the balloon for POT?**

The diameter of the balloon used for proximal optimization (POT) should correspond to the diameter of the proximal MV, which, based on Finet’s fractal law, should correspond to ~ ⅔ of the sum of the distal MB diameter and the SB diameter. Its length should be equal to or shorter than the MV stent while ensuring sufficient coverage to maintain at least 6 mm—ideally 8–10 mm—of stent extending proximally beyond the carina after deployment.

## 4. Second Stent Placement Following Provisional Approach: Step by Step Techniques

### 4.1. Steps for the T and Protrusion (TAP) Technique (Figure 5)

**Figure 5:**
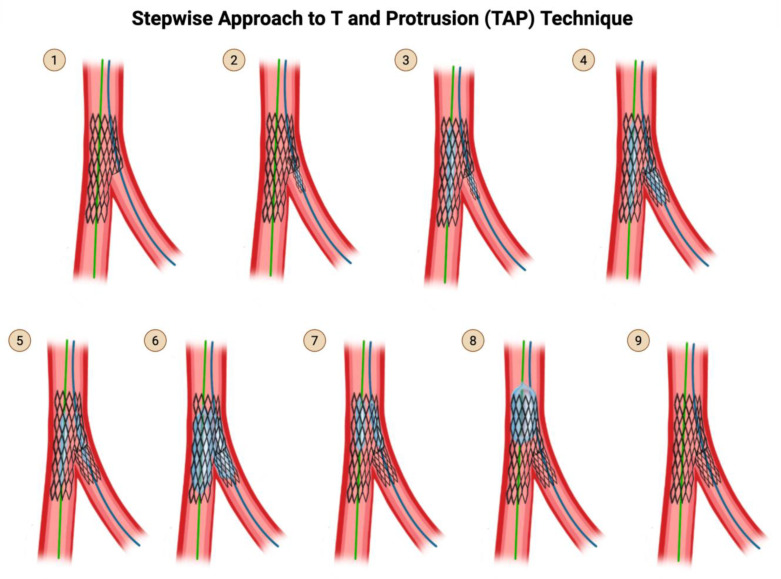
TAP Technique. Original illustration.

**Step 1:** This technique picks up from the last step of provisional stenting technique (see Step 9 of Figure 5), after you have rewired the SB and dilated through the MV stent struts with a small compliant balloon at high pressure.

**Step 2:** Advance a stent into the SB across the SB ostium, BUT do not deploy it yet.

**Step 3:** Advance a balloon sized 1:1 to the MV stent but shorter than the MV stent and position the center of the balloon at the carina of the bifurcation. Make sure that this balloon does not protrude outside of the original stent.

- The reason you cannot deploy your SB stent before first advancing a balloon in position in the MV is that a deployed stent in the SB may have protruding stent struts that prohibit the advancement of the main branch balloon.
- The MV balloon should be positioned and waiting, and the undeployed SB stent should be positioned and waiting.

**Step 4:** Pull back the SB stent such that it protrudes minimally (1–2 mm) into the MV. Deploy the SB stent. Perform an angiogram to verify that there is no distal dissection that would require the delivery of a second stent into the SB.

- Do not remove the wire from the SB because you have no plans for crushing and rewiring the SB stent—instead, perform kissing balloon inflation, so at no point does your SB become fenced off.

**Step 5:** Take the stent balloon of the already-deployed SB stent and slightly withdraw it further into the proximal MV, such that part of the balloon protrudes proximally from the SB stent. Align the proximal tip of the SB balloon with the proximal tip of the main branch balloon.

**Step 6:** Perform kissing balloon inflation with the SB stent balloon and the main branch balloon.

**Step 7:** Deflate both balloons, but make sure to deflate the main branch balloon first before deflating the SB balloon.

- If you mistakenly deflate the SB balloon before deflating the main branch balloon, you will crush the proximal stent struts of the SB into the SB ostium, which would then force you to convert to a reverse crush technique and have to rewire the fenced-off SB.
- Remove both balloons, paying attention so as not to lose the wire position.

**Step 8:** (Optional) Perform post-dilation of the proximal main branch for proximal optimization.

- The distal marker of the balloon used for POT should be positioned just proximal to the neocarina, because remember that now, your SB stent has created an artificial carina that is 1–2 mm more proximal than the original carina of the bifurcation.
- Beware that if you mistakenly advance the POT balloon beyond the neocarina, you will crush the protruding proximal struts of the SB stent. At this point, you may consider either to convert to a reverse crush technique and rewire the fenced-off SB or to reopen the partially crushed SB stent by performing another kissing balloon inflation, since there is still a wire in the SB through the SB stent lumen.
- For this reason, final POT is not a required step in TAP technique. To reduce the risk of POT ballooning beyond the neocarina, stent boost mode should be used to visualize the position of the balloon.

**Step 9:** Remove the balloon used for POT. Perform an angiogram for a final assessment.

**Practice points regarding TAP**

The advantage of TAP is that it does not require rewiring of the SB, which reverse crush requires. The drawback of TAP is that it creates a neocarina more proximal than the original carina, which may impede the ability to advance equipment into the MV. The position of the neocarina is determined by the angle of the SB takeoff and the site of strut crossing. There are several tips to consider while performing TAP. When the SB has a T-shape take-off (90°), only small or absent stent protrusion from the SB is needed to successfully cover the SB ostium. When the SB has a Y-shape take-off (acute angle, generally <60°), a longer, oval-shaped protrusion of the SB stent may be needed to adequately cover the SB ostium, creating a neocarina in the main branch. However, TAP may be Performed at narrow angle <60 while minimizing the protruding neocarina, by following the 4 tips indicated in Table 1, with the particular emphasis on crossing the distal MB stent struts upon rewiring the SB.

**Rewiring through a distal strut into the SB**

When rewiring through the main branch struts to re-enter the SB, in provisional SB ballooning, in the TAP technique, and the Culotte technique, it is preferable to cross through a distal strut, as close as possible to the carina, rather than through a proximal strut. Distal recrossing near the carina, followed by ballooning at that site, provides better scaffolding of the SB ostium. In contrast, ballooning through a proximal recrossing site can lead to the inward displacement of the stent struts toward the MV lumen. In contrast, crossing through a proximal strut or mid strut is preferable in the crush family of techniques.

**Figure 6:**
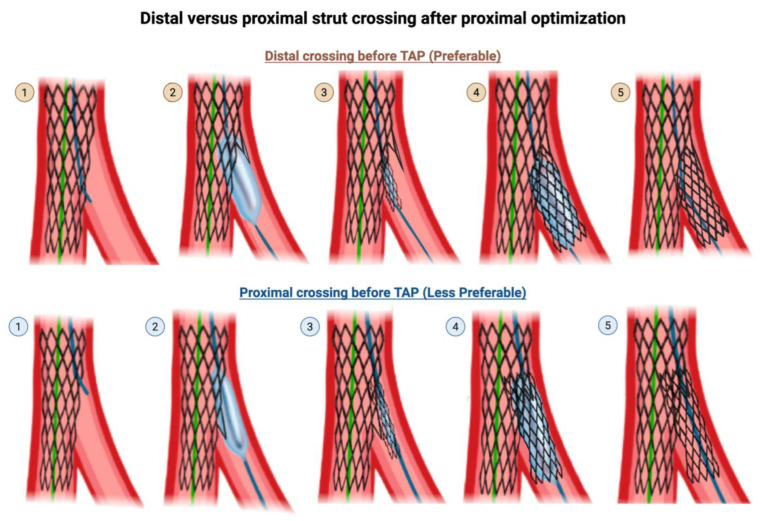
Wire crossing into the SB at a distal strut versus proximal strut prior to T-and-protrusion technique after provisional technique. **Top row**: (Step 1) Rewiring through the MV stent struts into the SB should be done through a distal cell. (Step 2) SB balloon dilation lifts the MB stent and allows it to scaffold the upper arm of the SB, such that subsequent (Step 3) delivery and (Step 4) deployment of the SB stent in TAP technique results in (Step 5) a final result with minimal protrusion of the SB stent into the MV. **Bottom row**: (Step 1) Rewiring through the MV struts should not be done through a proximal cell. (Step 2) SB balloon dilation deforms the MV stent at an angle that opens perpendicularly into the SB wall rather than into the SB lumen, such that subsequent (Step 3) delivery and (Step 4) deployment of the SB stent in TAP technique results in (Step 5) a final result with significant, undesired protrusion of the SB stent into the MV. This illustration is magnified for the reader to visualize the effects of distal versus proximal crossing. Original illustration.

When the wire crosses into the SB via a distal strut, SB balloon dilation lifts the MB stent and allows it to scaffold the upper arm of the SB, such that subsequent TAP stenting of the SB results in minimal protrusion into the MV (Figure 6). Conversely, when the wire crosses into the SB via a proximal strut, SB balloon dilation deforms the MV stent at an angle that opens perpendicularly into the SB wall rather than into the SB lumen, such that subsequent TAP stenting of the SB results in a significant protrusion into the MV. If the neocarina extends too far into the MB, TAP may be converted to a provisional mini-culotte or internal crush.

### 4.2. T Technique

**Figure 7:**
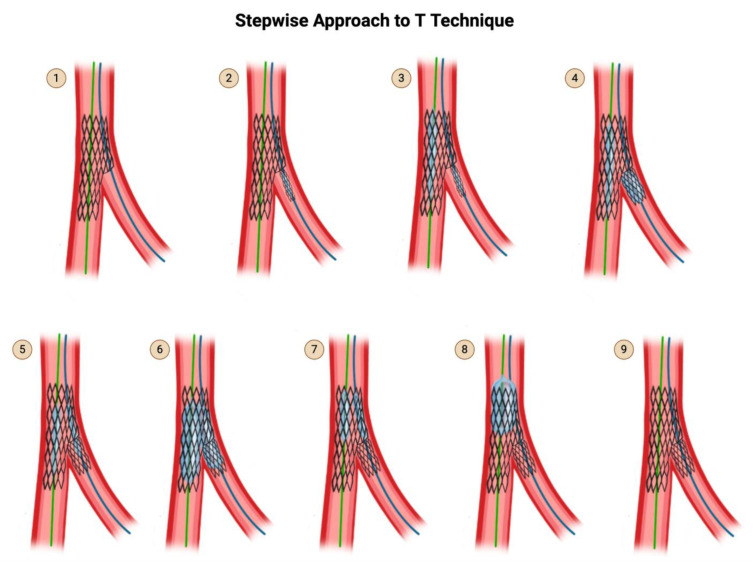
T Technique. Original illustration. Please note that the description for steps 1 through 9 are identical to that of TAP (Figure 5), except for step 4. In step 4, if the SB stent is deployed with no protrusion into the main branch, it is a T. If the SB stent is deployed more proximally with a 1–2 mm protrusion into the main branch, it is a TAP.

The T technique was the predecessor of the T and protrusion technique. The same steps are followed, but whether the result is T or TAP technique determines on how perfect the TAP technique was applied and how much the SB stent ends up protruding into the MV (Figure 7). If the MB struts are rewired through a distal strut, particularly with a favorable bifurcation angle (Table 1), the result is a SB stent deployed with no protrusion, which is a T. If the SB stent is deployed more proximally with a 1–2 mm protrusion into the main branch, it is a TAP [37].

### 4.3. Reverse Crush (Internal Crush) Technique

**Figure 8:**
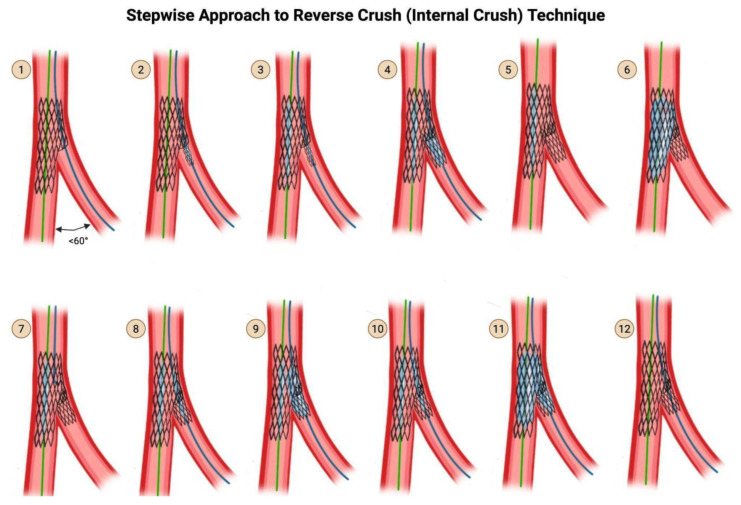
Reverse Crush (Internal Crush) Technique. Original illustration.

When the angle between the main branch and SB is acute, less than 60°, the use of the T and protrusion technique may cause excessive protrusion on one side as you attempt to fully cover the ostium of the SB. In this situation, the reverse crush technique (Figure 8) is an alternative.

**Step 1:** This technique picks up from the last step of provisional stenting technique (see Step 9 of Figure 6), after you have dilated through the MV stent struts with a small compliant balloon at high pressure.

**Step 2**: Advance a stent into the SB BUT do not deploy it yet.

**Step 3**: Advance a balloon sized 1:1 to the MV stent but shorter than the MV stent, and position the center of the balloon at the carina of the bifurcation. Make sure that this balloon does not protrude outside of the original stent.

- The reason you cannot deploy your SB stent before first advancing a balloon in position in the MV is that a deployed stent in the SB may have protruding stent struts that prohibit advancement of the main branch balloon.
- The undeployed balloon should be in position in the main stent and the undeployed stent in position in the SB.

**Step 4:** Withdraw the undeployed stent 1–2 mm into the main branch, and deploy the stent. Then remove the stent delivery system. Perform an angiogram to verify that there is no distal dissection that would require the delivery of a second stent into the SB.

**Step 5:** Remove the wire from the SB.

**Step 6:** Inflate the already-positioned main branch balloon at high pressure.

**Step 7:** Recross the SB stent’s struts with a wire.

**Step 8:** Advance a balloon down the recrossed SB, with the balloon sized to the SB reference diameter.

**Step 9:** Inflate the SB balloon at high pressure (12–20 atm), through the proximal struts of the SB stent.

**Step 10**: Align the proximal end of the SB balloon and the main branch balloon.

**Step 11:** Perform two-step kissing balloon inflation.

**Step 12:** Remove both balloons carefully. Perform an angiogram for the final assessment.

## 5. Two-Stent Intention-to-Treat Approach: Step by Step Techniques

There are several two-stent intention-to-treat techniques. Classic crush, mini-crush, nano crush, and DK crush can be thought of as a collection of variations on the same technique. Inverted Culotte and reverse Culotte can be thought of variations on the same technique. V and simultaneous kissing stent can be thought of variations on the same technique.

### 5.1. Classic Crush Technique (Figure 9)

**Step 1:** Wire the MV and the SB.

**Step 2 & 3:** Advance a balloon in the MV and perform pre-dilation of the MV.

**Step 4 & 5:** Advance a balloon in the SB and perform pre-dilation of the SB.

**Step 6:** Advance both the SB stent into the SB and the MV stent in the MV. Pull back the SB stent such that there is a 4–5 mm protrusion proximally into the MV.

**Step 7:** Deploy the SB stent first. Perform an angiogram to verify that there is no distal dissection that would require the delivery of a second stent into the SB. Now that you have stented the SB, remove the SB stent delivery system.

**Step 8:** Deploy the MV stent. This crushes the 4–5 mm proximal segment of the SB stent that was protruding into the MV.

**Step 9:** Remove the main branch wire and remove the SB wire. Perform an angiogram for final assessment.

In the original description of the classic crush technique in 2003, this was considered the end of the procedure [38]. Subsequent versions of crush (not illustrated here) introduced the concept of re-wiring the SB through the jailed struts and performing kissing balloon inflation. Subsequent versions also introduced the importance of post-dilation of the proximal segment of the main branch stent, known as proximal optimization technique, or POT. In modern day parlance, with the incorporation of kissing balloon inflation and POT (see subsequent illustrations), classic crush, mini-crush, and nano-crush are considered to be the same technique, varying only by the degree of SB stent protrusion: 4–5 mm for classic crush, 2–3 mm for mini-crush, and 0.5–1 mm for nano-crush.

### 5.2. The Mini-Crush Technique (Side-Branch Stent Crushed by the Main Branch Stent)

Recognizing that a 4- to 5 mm protrusion of the SB stent was not necessary and led to a large volume of crushed stent and thus greater stent deformity, the mini crush reduced the amount of protrusion from 4–5 mm to 2–3 mm [39]. This technique differs depending on whether 7 Fr or 6 Fr access is used. The 7 Fr technique is preferred over the 6 Fr technique as the 7 Fr technique provides more stability and allows for the advancement of two stent delivery systems together.

**Figure 10:**
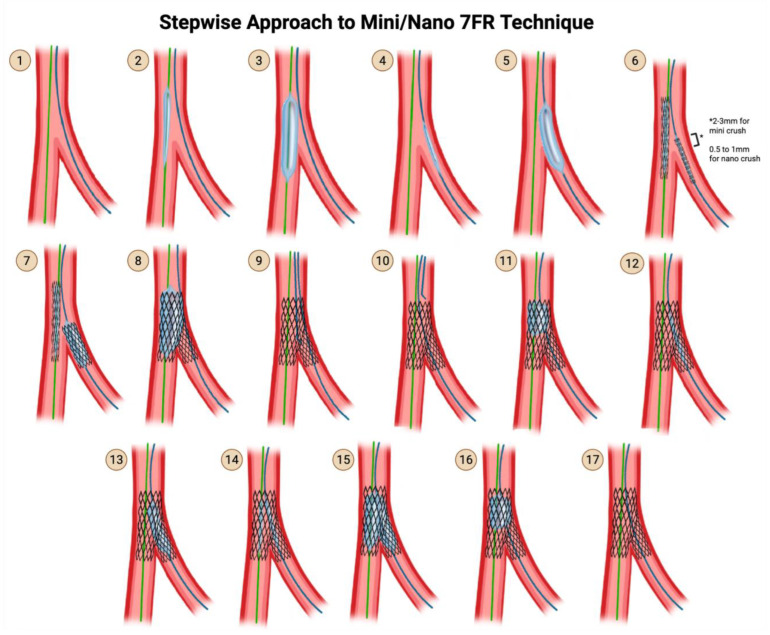
Mini/Nano Crush using 7 Fr Technique. Original illustration.

**Steps for mini crush using 7 Fr (Figure 10)**

**Step 1:** Wire the MV and the SB.

**Step 2 & 3:** Advance a balloon in the MV and perform pre-dilation of the MV.

**Step 4 & 5**: Advance a balloon in the SB and perform pre-dilation of the SB.

**Step 6:** Advance both the SB stent into the SB and the MV stent into the MV. Pull back the SB stent such that there is a 2–3 mm protrusion proximally into the MV.

**Step 7**: Deploy the SB stent first. Perform an angiogram to verify that there is no distal dissection that would require the delivery of a second stent into the SB. Now that you have stented the SB, remove the SB stent delivery system.

**Step 8:** Deploy the MV stent. This crushes the 1–2 mm proximal segment of the SB stent that was protruding into the MV.

**Step 9 & 10:** Rewire the SB through the struts of the MV stent and the crushed proximal struts of the SB stent. Rewire through non-distal struts (proximal of mid-struts), unlike in the provisional strategy and in culotte technique, where rewiring is performed through distal struts.

**Step 11:** Perform post-dilation of the proximal main branch for proximal optimization, using a balloon-sized 1:1 to the proximal MV.

**Step 12 & 13:** Advance a small-profile compliant balloon and perform high-pressure dilation through the struts of the MV stent into the SB.

**Step 14 & 15:** Now that you have an opening into the SB again, advance a non-compliant balloon into the SB and a non-compliant balloon into the MV, line up the proximal dots, and perform kissing balloon inflation.

**Step 16:** Finally, perform post-dilation once again of the proximal main branch for proximal optimization, using a balloon-sized 1:1 to the proximal MV.

**Step 17:** Perform an angiogram for a final assessment.

**Figure 11:**
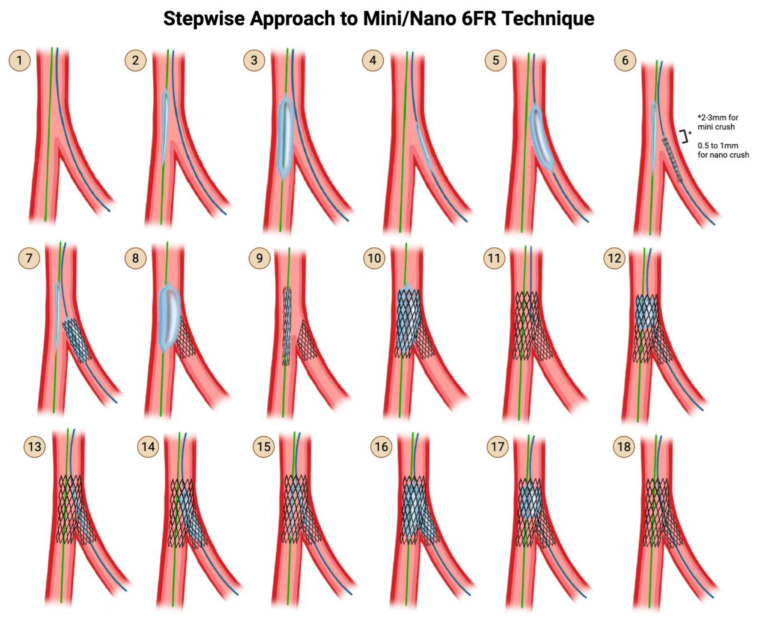
Mini/Nano Crush using 6 Fr Technique. Original illustration.

**Steps for mini crush using 6 Fr (Figure 11):**

- In a 6 Fr system, it is not possible to advance two stent delivery systems together. One stent delivery system has to be removed before the other stent delivery system can be advanced. Therefore, mini crush performed using a 6 Fr system has to be modified as follows.

**Step 1:** Wire the MV and the SB.

**Step 2 & 3:** Advance a balloon in the MV and perform pre-dilation of the MV.

**Step 4 & 5:** Advance a balloon in the SB and perform pre-dilation of the SB.

**Step 6:** Advance the SB stent into the SB and the MV balloon into the MV. Pull back the SB stent such that there is a 2–3 mm protrusion proximally into the MV.

Note that in the 7 Fr technique, you would be advancing stents into both the SB and MV, whereas in this 6 Fr technique, you are advancing a stent into the SB and a balloon into the MV.

**Step 7**: Deploy the SB stent first. Perform an angiogram to verify that there is no distal dissection that would require the delivery of a second stent into the SB. Now that you have stented the SB, remove the SB stent delivery system AND SB wire. Throughout this time, the MV balloon is waiting in place.

**Step 8:** Inflate the already-positioned MV balloon. This crushes the 1–2 mm proximal segment of the SB stent that was protruding into the MV.

**Step 9:** Remove the MV balloon and insert the MV stent.

**Step 10**: Deploy the MV stent.

- Note that the deployment of the MV stent is not responsible for crushing the 1–2 mm proximal segment of the SB stent, because it was already crushed by the MV balloon.

**Step 11:** Rewire the SB through the non-distal struts of the MV stent and the crushed proximal struts of the SB stent.

- Note that in the 7 Fr technique, you would be rewiring the crushed proximal struts of the SB stent immediately after crushing it, but in this 6 Fr technique, you cannot rewire it immediately, because you have to wait for the MV stent to be inserted and deployed, before you rewire the SB.

**Step 12:** Now that you have deployed both stents and rewired the SB, perform post-dilation of the proximal main branch for proximal optimization, using a balloon sized 1:1 to the proximal MV.

**Step 13 & 14:** Advance a small-profile compliant balloon and perform high-pressure dilation through the struts of the MV stent into the SB. Remove this balloon.

**Step 15 & 16:** Now that you have an opening into the SB again, advance a non-compliant balloon into the SB and a non-compliant balloon into the MV, line up the proximal dots, and perform kissing balloon inflation.

**Step 17:** Finally, perform post-dilation once again of the proximal main branch for proximal optimization, using a balloon sized 1:1 to the proximal MV.

**Step 18:** Perform an angiogram for final assessment.

### 5.3. The Nano Crush Technique

Nano crush refers to a modification of mini-crush where the proximal protrusion is only 0.5 to 1 mm instead of 2–3 mm. This may only be achievable when the bifurcation angle is close to 90°.

**Practice points regarding mini crush**

There is one major scenario where the crush family of techniques is advantageous compared to Culotte and TAP: the case of critical disease >90% in both the distal MB and SB, where the first stent deployment in one distal branch may result in occlusion of the other distal branch due to plaque shift. In the crush technique, constant balloon access is maintained to the other distal branch, whereas in Culotte and TAP, there is only jailed wire access to that other distal branch, and rewiring it is needed before being able to balloon it. Prolonged ischemic time of the occluded SB may result from difficulty rewiring. Thus, crush techniques are preferred in case of critical disease in both distal branches.

### 5.4. Step Crush and Double-Kissing (DK) Crush

**Figure 12:**
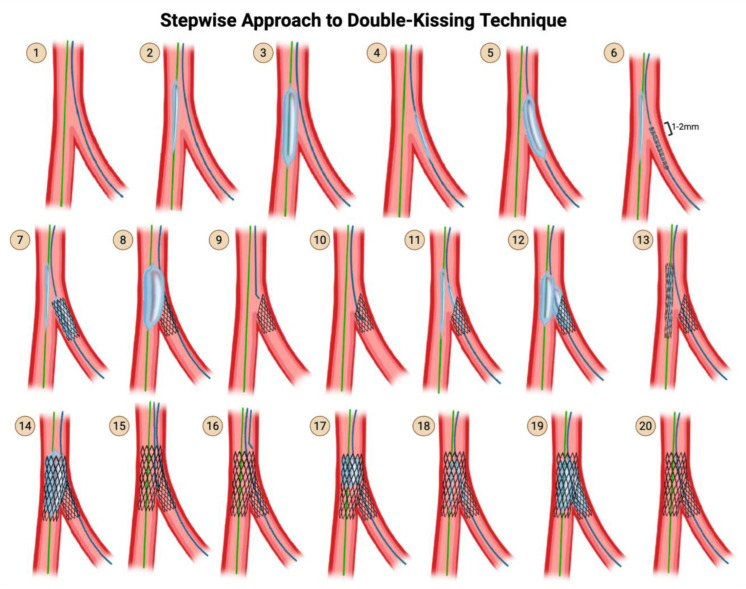
DK Crush Technique. Original illustration.

An additional kissing balloon inflation was added right after the deployment of the SB stent in order to clear the crushed struts of the SB stent away from the SB ostium, in order to increase the likelihood of successful recross after the subsequent MV stenting. This technique is thus called double kissing, or DK, because kissing balloon inflation happens twice: first, after deployment of the SB stent but before the insertion of the MV stent; second, after deployment of both stents and rewiring and strut-dilation of the SB, but prior to the final POT (Figure 12). In contrast, single kiss techniques refer to classic crush, mini crush, and nano crush. Although the initial description of DK crush used 4–5 mm of proximal protrusion of the SB stent, contemporary DK crush uses 2–3 mm proximal protrusion, just like how the 4–5 mm proximal protrusion of classic crush evolved into the 2–3 mm proximal protrusion of mini crush.

**Step 1:** Wire the MV and the SB.

**Step 2 & 3:** Advance a balloon in the MV and perform pre-dilation of the MV.

**Step 4 & 5:** Advance a balloon in the SB and perform pre-dilation of the SB.

**Step 6:** Advance both the SB stent into the SB and the MV balloon into the MV. Pull back the SB stent such that there is a 2–3 mm protrusion proximally into the MV.

**Step 7:** Deploy the SB stent first. Perform an angiogram to verify that there is no distal dissection that would require the delivery of a second stent into the SB.

- Remove the stent delivery system from the SB.

**Step 8:** Inflate the balloon that has been sitting waiting in the MV, thereby crushing the proximal edge of the freshly deployed SB stent.

- Remove the MV balloon.

**Step 9–10:** Rewire the SB through the struts of the MV stent and the crushed proximal struts of the SB stent.

**Step 11–12:** Advance a small-profile compliant balloon through the struts of the SB stent, and advance a balloon into the MV. Perform simultaneous kissing balloon inflation. Remove both balloons.

**Step 13–14:** Advance a stent into the MV and deploy it.

**Step 15:** Rewire the SB through the non-distal struts of the MV stent.

- In DK crush, the attempt to rewire the SB only has to cross through 1 layer of stent—the MV stent—because the proximal crushed struts of the SB stent were already opened up in the first round of kissing balloon inflation.
- In contrast, in classic crush and mini crush, the attempt to rewire the SB requires the wire to cross both the MV stent struts and the crushed SB stent struts.

**Step 16:** Perform post-dilation of the proximal main branch for proximal optimization, using a balloon sized 1:1 to the proximal MV.

**Step 17:** Use a small-profile compliant balloon and perform high-pressure dilation through the struts of the MV stent into the SB.

**Step 18:** Advance a non-compliant balloon into the SB and a non-compliant balloon into the MV, line up the proximal dots, and perform kissing balloon inflation.

**Step 19:** Finally, perform post-dilation once again of the proximal main branch for proximal optimization, using a balloon sized 1:1 to the proximal MV.

**Step 20:** Perform an angiogram for a final assessment.

**Practice points regarding DK crush**

The DKCRUSH-I trial of 2006 demonstrated that final kissing balloon inflation could be successfully performed in 100% of DK crush cases compared with 75% of classic crush cases, and that this translated into reduced MACE and improved target lesion revascularization-free survival. Successful final kissing balloon inflation is much higher in mini-crush (up to 98% in registries) than classic crush (75%), but probably less than in DK crush.

### 5.5. The Culotte Technique

Culotte is appropriate for Y-shaped angulated bifurcations. It is traditionally said that Culotte can only be performed if there are no vessel size differences between the main and SB. However, this is a myth. There is almost always going to be a vessel mismatch between MB and SB, as per Finet’s fractal law. Moreover, it is widely known that stents may be expanded beyond their nominal sizes, as is performed with POT and as shown by the specific manufacturers’ stent charts. Therefore, it would be more accurate to state that Culotte cannot be performed if there is a major vessel mismatch between MB and SB exceeding 1.5 mm.

The major drawback of the Culotte technique is that it leads to an excess of metal covering the proximal end, as there are two full layers of stent in the proximal part of the MV. The advantage, of course, is that Culotte gives the fullest coverage of the carina. The inverse culotte technique involves initial SB stenting followed by main branch stenting. Reverse culotte refers to initial main branch stenting, followed by SB stenting.

**Figure 13:**
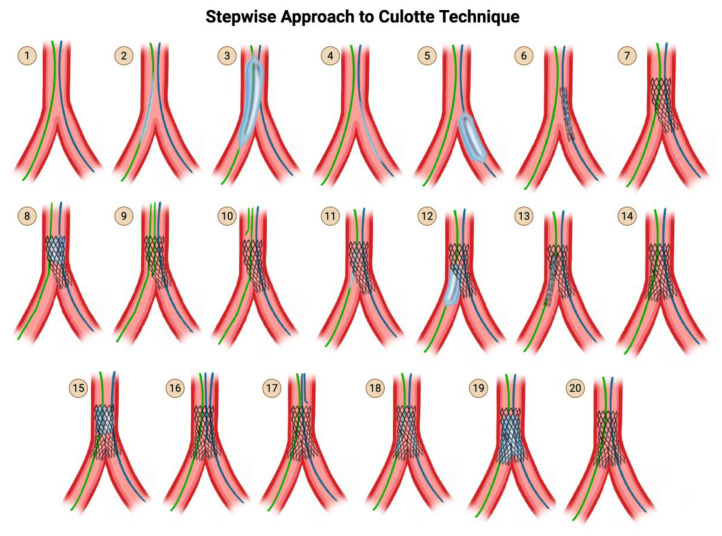
Culotte Technique. Original illustration.

With regard to which branch to stent into first, since there may be ambiguity about which branch should be designated as the SB, the first stent is traditionally placed from the proximal MB into the most angulated SB and/or the most critical SB. In that case, the distal MB may be referred to as the assigned SB. This is referred to as inverted Culotte. Alternatively, if the first stent is placed from the proximal MB into the distal MB, it is referred to as reverse Culotte. In the steps that follow (Figure 13), inverted Culotte is depicted.

**Steps for Culotte and DK Culotte:**

**Step 1:** Wire the MV and the SB.

**Step 2 & 3:** Advance a balloon to the MV and perform pre-dilation of the MV.

**Step 4 & 5:** Advance a balloon to the SB and perform pre-dilation of the SB.

**Step 6:** Advance the SB stent into the SB. Position the SB stent such that the proximal portion covers the entirety of the diseased segment of the proximal main branch and the distal portion covers the entirety of the diseased segment in the SB.

- Note that this is in contrast to mini crush, where you only pull back just enough such that there is a 2–3 mm protrusion proximally into the MV.

**Step 7:** Deploy the SB stent.

- This assumes that the SB is more angulated. If the main branch is more angulated than the SB, then you should treat the main branch as the SB throughout the entire procedure and perform stenting of that first.

Perform an angiogram to verify that there is no distal dissection that would require the delivery of a second stent into the SB. Remove the SB stent delivery system, but not the SB wire.

**Step 8:** Perform post-dilation of the proximal main branch for proximal optimization, using a balloon-sized 1:1 to the proximal MV. This further jails the MV wire.

- Note that this is the first POT.

**Step 9:** Rewire the distal MV using a new wire, through the struts of the freshly placed stent in the SB extending back to the proximal MV. Rewire the stent struts distally, similarly to provisional stenting.

**Step 10:** Once you have rewired the distal MV, you can remove the jailed MV wire.

**Step 11 & 12:** Use a small-profile compliant balloon and perform high-pressure dilation through the struts of the SB-proximal MV stent into the distal MV.

- (Optional) Kissing balloon inflation may then be optionally performed, in which case culotte becomes DK culotte. This is not depicted. This kissing balloon inflation may facilitate rewiring of step 16.

**Step 13:** Then insert the MV stent such that part of it protrudes into the distal MV past the carina, and a significant portion of it overlaps with the first stent in the proximal MV.

**Step 14:** Deploy the MV stent. Remove the stent delivery system from the main branch, but not the main branch wire.

**Step 15:** Perform post-dilation of the proximal main branch for proximal optimization, using a balloon sized 1:1 to the proximal MV. This further jails the SB wire. Note that this is the second POT.

**Step 16–17:** Rewire the SB through the struts of the MV stent. Once you have rewired the SB, you can remove the jailed SB wire.

**Step 18:** Advance a non-compliant balloon into the SB and a non-compliant balloon into the MV, line up the proximal dots, and perform kissing balloon inflation.

- Inflate only the SB balloon at high pressure.
- Then inflate only the main branch balloon at high pressure.
- In Culotte, it does not hugely matter which balloon inflation happens first, so it is OK to perform main branch balloon inflation before SB balloon inflation.
- Now that you have individually inflated each balloon, simultaneously inflate both the main branch balloon and SB balloon at 12–14 atm.
- Deflate both balloons at the same time.
- Remove both balloons.

**Step 19:** Finally, perform post-dilation once again of the proximal main branch for proximal optimization, using a balloon sized 1:1 to the proximal MVl.

- In Culotte, this final and third POT is optional, since you have already performed POT twice earlier in the procedure. Therefore it is not illustrated in the diagram.

**Step 20:** Perform an angiogram for final assessment.

### 5.6. The V and the Simultaneous Kissing Stent Techniques

**Figure 14:**
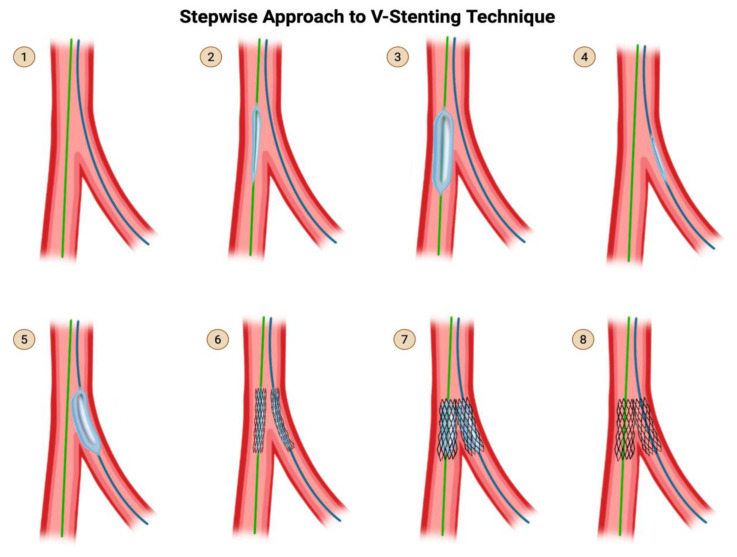
V-Stent Technique. Original illustration.

V stenting can only be used for Medina (0,1,1) bifurcations, i.e., bifurcations with disease in the distal main branch and SB, with no significant disease in the proximal MV. The drawback of V-stenting is the formation of a neocarina from the new stents that can hinder the ability to advance equipment in the future. The advantage of V-stenting is its simplicity, in that there is no rewiring needed and no kissing balloon inflation needed. You will never lose access to either of the branches. It is simple but it is not elegant. Because it requires simultaneous delivery of both stents, V-stenting can only be performed in a 7-Fr system (Figure 14).

**Steps for V-stenting:**

**Step 1:** Wire the MV and the SB.

- Note that in V-stenting, you are treating the SB and the distal MV symmetrically, such that you can place two stents in a V-shape.
- Therefore, from here onward, I will refer to the SB and the distal MV both as distal “branches”.

**Step 2 & 3:** Advance a balloon in the main branch (taking care to reduce balloon involvement of the healthy proximal segment) and perform pre-dilation.

**Step 4 & 5**: Advance a balloon in the SB (taking care to reduce balloon involvement of the healthy proximal segment) and perform pre-dilation.

**Step 6:** Advance one stent each into each distal branch.

- There should not need to be significant overlap in the proximal vessel because this technique is not supposed to be used if there is significant proximal vessel disease.

Adjust the position of both stents such that they are protruding only 1–2 mm into the proximal vessel.

**Step 7:** Simultaneously inflate both stents. Take care to deflate both stents simultaneously as well. Then remove both stent delivery systems.

**Step 8:** Perform an angiogram for final assessment.

**Simultaneous Kissing Stent technique:**

**Figure 15:**
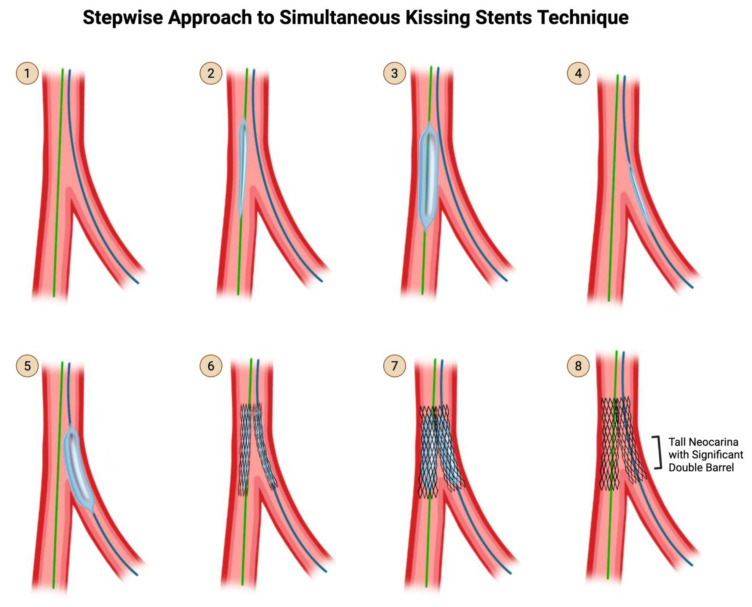
SKS Technique. Original illustration. Please note that the description for steps 1 through 8 are identical to that of V-stenting (Figure 14), except for step 6. In step 6, if the protrusion into the proximal MV is only 1-2mm, it is V-stenting. If the protrusion into the proximal MV is significantly more than 1-2mm, it is SKS, creating a tall neocarina with a significant double barrel.

Simultaneous kissing stent technique is the exact same thing as V-stenting except the protrusion into the proximal vessel is significantly more than 1–2 mm, creating a significant double barrel segment of stent in the proximal vessel (Figure 15). The biggest disadvantage of SKS is the creation of a long neocarina unnecessarily in the proximal vessel, which will make subsequent equipment delivery unnecessarily challenging. SKS should only be used in an emergency or for bailout when there are no other two-stent techniques that can be performed in time. Simultaneous kissing stent should not be confused with simultaneous kissing balloon inflation, which is part of almost every technique.

### 5.7. The “Y” and the “Skirt” Techniques

This technique is more of historical interest than contemporary practice, as it was the first bifurcation stenting technique demonstrated in a live case course. In the Y-stent or skirt technique, after pre-dilation of each branch, a stent is deployed in each branch with minimal to no protrusion into the proximal MV. If the results are not adequate, than a third stent is deployed in the main branch, giving the final piece of the Y shape. In order to expand and approximate the proximal stent to the already-deployed stents, two balloons are advanced into the third stent that was freshly placed in the proximal MV. The two balloons are positioned such that the distal part of each balloon sits in each of the distal branches. The two balloons are then simultaneously inflated, causing the distal segment of the third stent to “skirt” out, allowing it to approximate the two distal stents.

## 6. MADS Classification

The MADS classification, proposed in 2008 by the European Bifurcation Club, provides an approach of defining and grouping coronary artery bifurcation stenting strategies based on the final position of the stent(s) and the implantation order.

Unlike the widely used Medina classification, which is focused on categorization of lesions, MADS focuses on procedural stent deployment strategies.

**Figure 16:**
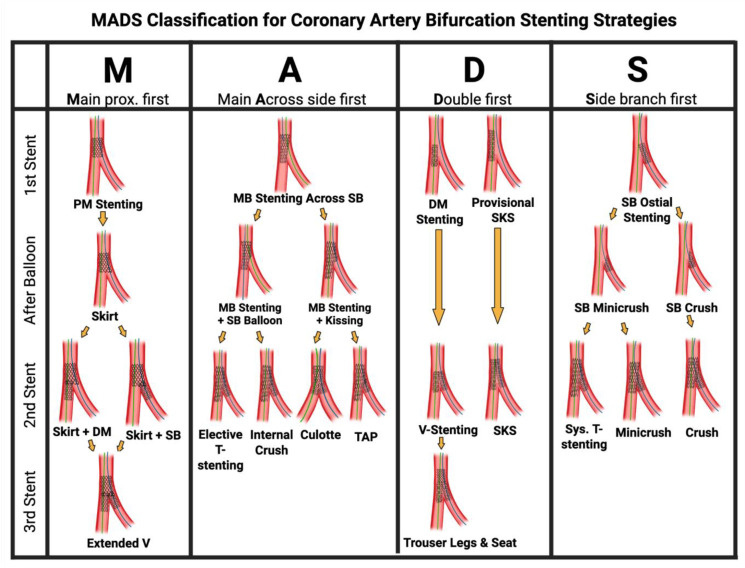
The MADS classification for defining and grouping coronary artery bifurcation stenting strategies based on the final position of the stent(s) and the implantation order. Original illustration.

The position of the first stent in a bifurcation is designated by one of the four MADS strategies [18] (Figure 16). M (Main) refers to techniques that begin with the proximal main segment, stenting the MV prior to treating the SB. A (Across) refers to techniques that begin with a stent in the MV across the side. D (Distal/Double) refers to techniques that involve double stent implantation, simultaneous or not. S (SB first) refers to techniques that begin with a stent in the SB, with protrusion or not. This is usually employed when preserving SB access is critical due to high-risk occlusions. Following the first stent(s), additional stents may be placed using different techniques.

## 7. Conclusions

The treatment of bifurcation coronary artery disease may be challenging, requiring a structured and individualized approach. Accurate lesion assessment using established bifurcation classification systems, SB evaluation, is essential for guiding the treatment strategy between a provisional stenting strategy or an upfront two-stent technique. Familiarity and understanding the steps of various bifurcation percutaneous coronary intervention techniques, as detailed in this manuscript, allows operators to make informed decisions tailored to lesion complexity and patient-specific factors. In Part 2 of this series [9], we will expand the discussion to include SB assessment, pressure wire guidance, comparative outcomes, and evolving strategies for individualized technique selection.

## Figures and Tables

**Figure 9:**
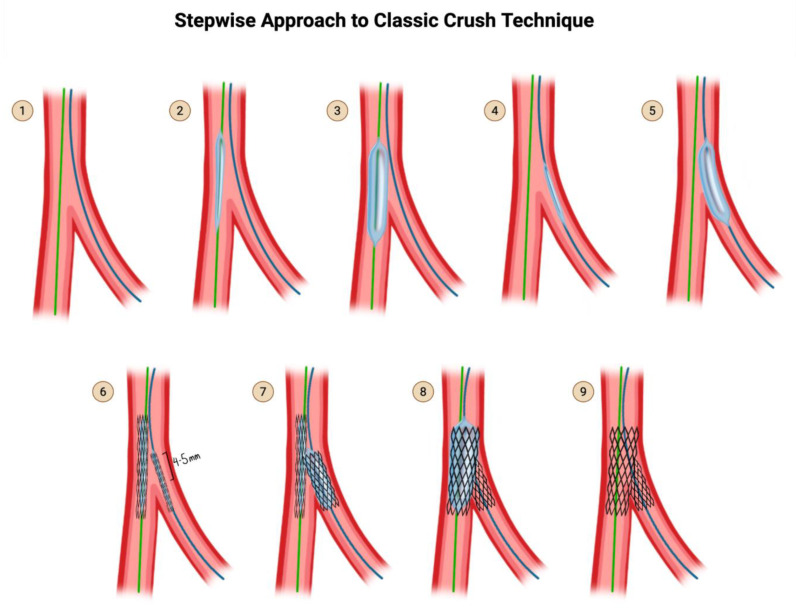
Classic Crush Technique, as originally proposed. Subsequent versions of crush (not illustrated here) introduced the concept of re-wiring the SB through the jailed struts and performing kissing balloon inflation, as well as proximal optimization. Original illustration.

**Table 1:** Tips for a successful TAP with minimal or no protrusion, particularly in narrow angles <60°. Reproduced with permission from Dr Elias Hanna.

(i) Perform proper main branch POT, which lifts some of the main branch stent struts into the ostium of the SB
(ii) Rewire SB through distal stent struts. This allows SB balloon dilatation to lift the MB stent and scaffold the upper arm of the SB, the one most difficult to cover
(iii) Use stent boost technology to minimize protrusion while avoiding the ostial miss
(iv) Consider using a “balloon-assisted” TAP technique: inflate a balloon inside the MB while positioning the SB stent, then pull the SB stent until it meets resistance from the inflated MB balloon, then deflate that balloon and deploy the SB stent
